# Clinical Remission and Its Determinants in Adult Severe Asthma Patients Receiving Biologic Therapy: A Retrospective Analysis

**DOI:** 10.3390/jcm15020442

**Published:** 2026-01-06

**Authors:** Dane Ediger, Esma Nur Aktepe Sezgin, Raziye Tülümen Öztürk, Burcu Çoban

**Affiliations:** Department of Pulmonary Diseases, Division of Allergy and Immunology, Faculty of Medicine, Bursa Uludag University, 16059 Bursa, Türkiye; dr.esmaktepe@gmail.com (E.N.A.S.); rtulumenozturk@uludag.edu.tr (R.T.Ö.); drburcucoban@hotmail.com (B.Ç.)

**Keywords:** severe asthma, biologic agent, mepolizumab, omalizumab, remission

## Abstract

**Background/Objectives:** In recent years, the concept of clinical remission under treatment in asthma has gained increasing attention. It is defined as the absence of exacerbations, asthma symptoms, and oral corticosteroid use for at least 12 months, together with improved or stable lung function. This study aimed to evaluate the clinical remission rates and associated factors in patients with severe asthma receiving biologic therapy with either omalizumab (anti-IgE) or mepolizumab (anti-IL-5). **Methods**: Adult patients with severe asthma and type 2 inflammation who started omalizumab or mepolizumab between January 2009 and December 2023 in our allergy clinic were retrospectively analyzed. Sociodemographic and clinical characteristics were reviewed. Clinical remission rates were assessed at the first and most recent years of maintenance therapy. Independent markers were identified using multivariable analyses. **Results**: A total of 160 patients were included (mean age 53.8 ± 14.6 years; 81.9% female). Of these, 85.6% received omalizumab and 14.4% mepolizumab. Remission rates at one year and at the latest follow-up were 60.0% and 43.7%, respectively. Patients achieving remission had higher total IgE levels. Psychiatric comorbidity negatively affected remission. The one-year remission rates were 91.3% in the mepolizumab group and 54.7% in the omalizumab group. Higher baseline blood eosinophil counts and Asthma Control Test (ACT) scores were positive markers, while psychiatric disease was inversely associated. **Conclusions**: Omalizumab and mepolizumab achieved meaningful clinical remission rates in severe asthma. Elevated ACT scores and eosinophil counts and absence of psychiatric comorbidities were independent markers, underscoring the need for individualized biologic therapy to achieve sustained remission.

## 1. Introduction

Asthma is a heterogenous disease characterized by chronic airway inflammation, defined by the presence of variable expiratory airflow and a history of respiratory symptoms such as wheezing, shortness of breath, chest tightness, and coughing, that fluctuate in timing and severity [1]. The European Respiratory Society (ERS)/American Thoracic Society (ATS) and the Global Initiative for Asthma (GINA) define difficult-to-treat asthma has modifiable factors are not optimized and can achieved control after optimization of treatment, adherence, inhaler technique, and management of comorbidities and also severe asthma as asthma that can only be controlled with high-dose inhaled corticosteroids and a second controller agent (e.g., long-acting beta agonist) after ensuring proper diagnosis, good inhaler technique, and high adherence, or asthma that cannot be controlled without these treatments [1,2]. Approximately 3–10% of asthma patients fall into the severe asthma category; this small group accounts for a disproportionately large portion of morbidity and healthcare resource utilization [2].

In severe asthma, biological therapies have been introduced into clinical practice since the early 2000s with the aim of reducing corticosteroid dependence, controlling exacerbations, and improving quality of life. However, the concept of clinical remission in severe asthma has emerged and become one of the focal points of research. However, the concept of remission in asthma does not yet have a single universally accepted definition [3]. Furthermore, the likelihood of clinical remission in asthma is influenced by many factors, such as the phenotype and endotype characteristics of the disease, initial eosinophil levels, the presence of comorbidities, treatment adherence, and the type of biological agent used [4,5,6,7,8,9].

The purpose of this study is to determine the clinical remission rates achieved with omalizumab and mepolizumab therapies in patients with severe asthma and to discover the factors that influence remission. Based on real-world data, this evaluation aims to contribute to understanding in which patient profiles biological treatments achieve higher success rates.

## 2. Materials and Methods

### 2.1. Study Design

**Figure 1:**
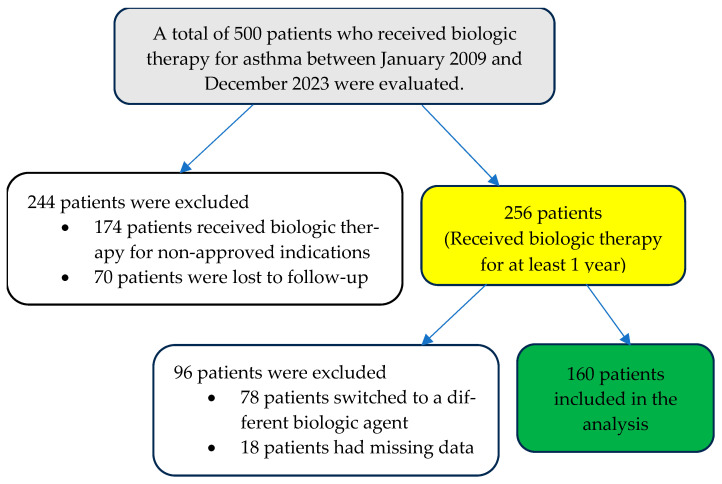
Flow chart of patients included and excluded from the study.

The study population consisted of adult patients diagnosed with severe type-2 asthma and initiated on biological therapy at the Allergy Outpatient Clinic of Bursa Uludag University Faculty of Medicine between January 2009 and December 2023. Before initiating biological agents, difficult-to-treat asthma was excluded, and biological treatment was initiated only in patients with severe asthma. Patient records were retrospectively reviewed. Patients who used the biological agent for less than a year, did not attend follow-up meetings on a regular basis, provided inadequate data, or switched to another biological agent were excluded from the research (Figure 1).

### 2.2. Ethical Approval

The local ethics committee approved the study (7 February 2024/Decision No: 2024-1/3). The study followed the ethical principles specified in the Declaration of Helsinki. All patients were treated and admitted in accordance with good clinical practice recommendations.

### 2.3. Data Collection

The study data were gathered by retrospectively evaluating patient files in the hospital’s computer records. Patients’ age, gender, body mass index (BMI), smoking status, amount of cigarettes smoked, accompanying atopy, chronic rhinosinusitis with and without accompanying nasal polyps, respiratory diseases exacerbated by nonsteroidal anti-inflammatory drugs (NERD), obstructive sleep apnea syndrome (OSAS), obesity (BMI ≥ 30 kg/m^2^), gastroesophageal reflux (GER), psychiatric disorders (anxiety, depression, bipolar disorder), and other chronic diseases (bronchiectasis, diabetes, hypertension, osteoporosis, immunodeficiency), age of asthma onset, duration of asthma, type of asthma inflammation (Allergic non-eosinophilic, nonallergic eosinophilic, allergic eosinophilic), type of biological agent used (omalizumab or mepolizumab), and treatment durations were examined and recorded for the study. The presence of atopy was determined by skin prick testing for dust mites, cockroaches, mold, cats, latex, pollen allergen extracts, and/or serum allergen-specific IgE positivity. The Asthma Control Test (ACT) score, the presence and number of attacks, the need for and number of systemic corticosteroid treatments, the need for maintenance steroids and the steroid withdrawal rate after biological treatment, hospital admissions and their number, respiratory function tests, serum total IgE, and peripheral eosinophil count were recorded.

### 2.4. Definitions

#### 2.4.1. Definitions for Asthma and Its Management

Severe asthma is asthma that can only be controlled with high-dose inhaled corticosteroids and a second controller agent (e.g., long-acting beta agonist) or that cannot be controlled without these treatments, and thus requires the addition of a biological agent as recommended in GINA Step 5. The appropriateness and application protocols of omalizumab and mepolizumab treatments were evaluated within the framework of current guidelines in type-2 asthmatics. Type-2 asthma includes allergic and/or eosinophilic asthma. Omalizumab was initiated in patients with perennial allergies and severe asthma who had Total IgE levels between 30 and 1500. The dose and frequency of omalizumab medication were calculated using a standard scale based on the patient’s body weight and serum total IgE level. Using this scale, patients were given subcutaneous omalizumab at an estimated dose of 150–600 mg every 2 or 4 weeks.

A blood eosinophilia ≥ 150 cells/µL cut-off is accepted for the diagnosis of eosinophilic asthma. In accordance with the reimbursement conditions of our healthcare system, mepolizumab was administered to severe eosinophilic asthmatics with blood eosinophilia ≥ 300 cells/µL and was given subcutaneously in a regular dose of 100 mg every four weeks.

An asthma attack is defined as the onset of symptoms such as increased shortness of breath, coughing, wheezing, or chest tightness in an asthmatic patient, accompanied by impaired respiratory function test results such as decreased peak expiratory flow (PEF) and forced expiratory volume in 1 s (FEV_1_), and the need for additional oral corticosteroids (OCS) treatment for improvement, as well as hospitalization or an emergency room visit.

#### 2.4.2. Definition of Remission

The primary endpoint of this study was defined as achieving clinical remission in severe asthma under treatment. Clinical remission under treatment was defined as meeting all the following criteria: no exacerbations for at least 12 months, no use of systemic corticosteroids for disease control, very low symptom burden or symptom-free status, and optimized or stabilized lung function. Failure to meet any of the specified clinical remission criteria was classified as non-remission. Full remission was defined as meeting the clinical remission criteria plus improvement in inflammatory markers from baseline [decrease in serum or sputum eosinophil count, decrease in fractional exhaled nitric oxide (FeNO) and other inflammation markers] and resolution of bronchial hyperreactivity. However, achieving complete remission was not defined as a goal or primary endpoint in this study; it was only distinguished as a conceptual definition.

Optimization of lung function was defined as post-bronchodilator FEV_1_ ≥ 80% predicted. Stabilisation of lung function was defined as a change in post-bronchodilator FEV_1_ not greater than a 5% decline from the baseline [10]. The stabilisation of respiratory function in severe asthma has not been fully defined. Given that McDowell et al. [11] demonstrated no meaningful differences in remission rates or associated clinical characteristics when using −100 mL, −5% proportional decline, or baseline FEV_1_ as cut-points, in this study, we defined lung function stabilisation as a ≤5% reduction in FEV_1_ from baseline for the purpose of remission classification.

ACT was used to confirm the absence of asthma symptoms. According GINA guideline, ACT scores range from 5–25 (higher is better). Scores of 20–25 are classified as ‘well controlled’, 16–19 as ‘not well-controlled’, and 5–15 as ‘very poorly controlled’ asthma. The ACT has four symptom/reliever questions plus patient self-assessed control. The minimum clinically important difference is 3 points. An ACT score of ≥20 was considered to indicate well-controlled asthma [1].

### 2.5. Statistical Analysis

The data was statistically analyzed using SPSS version 30.0 (IBM Corp., Armonk, NY, USA). Descriptive statistics provide mean ± SD values for continuous variables and frequency and percentage distributions for categorical variables. All analyses used a two-tailed *p*-value of <0.05 to indicate statistical significance. The distribution properties of the variables were assessed using the Kolmogorov-Smirnov test and skewness-kurtosis coefficients. Continuous variables that matched the normal distribution assumption were tested using parametric tests, whilst those that did not were tested using non-parametric methods.

When comparing groups, an independent samples t-test was used for continuous variables with a normal distribution, whereas the Mann–Whitney U test was employed for continuous variables that did not have a normal distribution. Pearson’s chi-square test was used in cross-tabulation analyses, Fisher’s exact chi-square test when applicable, and the Fisher–Freeman–Halton test for bigger tables to assess the connection between categorical variables. In comorbidity analyses, multiple testing correction was applied using the Holm–Bonferroni method for a priori defined comorbidities. In analyses involving a limited number of patients and the presence of outliers in chi-square tests, effect size was assessed using Cramer’s V. To evaluate the robustness of the results, confidence intervals were calculated using the bootstrap bias-corrected and accelerated (BCa) method (5000 resamples)

In multivariate studies, binary logistic regression was used to identify the independent factors that influence remission. The regression model contained variables that were statistically significant in univariate studies and clinically useful. The results were presented as odds ratios (OR) and 95% confidence intervals (CI).

## 3. Results

The study involved 160 patients. The patients’ average age was 53.76 years (standard deviation: 14.62), and 131 (81.9%) were female. Of the patients, 137 (85.6%) were receiving omalizumab treatment and 23 (14.4%) were receiving mepolizumab treatment. Clinical remission rates under biological therapy were 60.0% in the first year and 43.7% in the last year. There were no significant variations in age, gender distribution, BMI, disease duration, smoking status, or smoking amount among patients with remission (*p* > 0.05).

First-year assessment of clinical remission under biological therapy (Table 1); the median ACT prior to initiation of biological agents was [14 (12.5–16)] in patients in remission, which was significantly higher than in those not in remission [12 (10–15)] (*p* < 0.001). No significant differences were observed between the remission and non-remission groups in terms of pre-treatment attacks, OCS use, and number of hospitalizations (*p* > 0.05). When laboratory parameters were examined, the median (IQR) serum total IgE value was significantly higher in those in remission [138 (65.5–320.5) IU/mL] than in those not in remission [94.5 (45–186.5) IU/mL] (*p* = 0.048). There was no significant difference in peripheral blood eosinophil count before therapy and remission status (*p* = 0.129). Respiratory function test parameters (FEV_1_, FVC, FEV_1_/FVC) were similar in patients with and without remission (*p* > 0.05). The number of patients with stable and increased FEV_1_ changes was significantly higher in the remission group compared to the non-remission group (*p* > 0.001). Among patients who did not achieve remission, both the atopy rate and the proportion of patients with mite sensitivity were significantly higher compared to the atopy and mite sensitivity rates in patients who achieved remission (*p* = 0.022, *p* = 0.044). Based on phenotypic characteristics, all eight patients (8/8) with non-allergic eosinophilic asthma achieved remission and this group showed a significantly higher remission rate compared to other inflammation types (*p* = 0.021). The association between inflammatory phenotypes and remission status showed a Phi coefficient and Cramer’s V of 0.220. Bootstrap analysis based on 5000 resamples yielded a standard error of 0.048 and a bias of 0.008. The BCa 95% confidence interval ranged from 0.134 to 0.342, based on 160 valid cases.

Comorbidities were examined in relation to clinical remission status during the first year of maintenance biological therapy (Table 1). Psychiatric illness was observed more frequently in patients who did not achieve remission at either time point or maintained a significant association with remission status even after Holm–Bonferroni multiple testing correction. In the first-year assessment, psychiatric illness was detected in 46.9% of patients who did not achieve remission and in 16.7% of patients who achieved remission, indicating that the presence of psychiatric illness negatively affected remission rates (*p* = 0.001). Osteoporosis was observed in 9.4% of patients who achieved remission and 23.4% of patients who did not achieve remission and showed an inverse relationship with remission in unadjusted analyses (*p* = 0.027). None of the four patients with immunodeficiency achieved remission, and this difference was statistically significant in unadjusted analyses (*p* = 0.024). Osteoporosis and immunodeficiency were not determined a priori and lost their significance after multiple testing correction; therefore, they were considered exploratory findings. A priori comorbidities subject to multiple testing adjustment included nasal polyps, chronic rhinosinusitis, N-ERD, psychiatric illness, obstructive sleep apnea, gastroesophageal reflux, and bronchiectasis.

In the multivariate regression analysis, eight variables were evaluated: age, gender, BMI, FEV_1_%, ACT, psychiatric illness, serum total IgE level, and peripheral blood eosinophil count. Eosinophil counts were analyzed in successive 100 cells/µL intervals. The relationship between patients’ pre-treatment ACT level, eosinophil count, and presence of psychiatric illness and achieving clinical remission in the first year was evaluated using binary (binomial) logistic regression analysis (Table 2). The analysis revealed that these variables were significantly associated with remission. The presence of psychiatric illness was identified as the strongest independent risk factor. The likelihood of achieving remission in individuals with psychiatric illness was approximately 4.8 times lower than in those without (OR = 4.763; *p* < 0.001). A high ACT score prior to treatment significantly increased the likelihood of achieving remission (OR = 1.247; *p* < 0.001). This finding indicates that better asthma control at baseline positively influenced treatment response. Each 100 cells/µL increase in eosinophil count was significantly associated with increased odds of the outcome (OR = 1.142; *p* = 0.03). This finding suggests that higher baseline eosinophil levels may influence the treatment outcome.

After presenting our first-year remission results, we also evaluated the clinical remission of patients undergoing biological therapy in their most recent year to understand how these rates progressed over a longer period (Table 3). The median (IQR) ACT score prior to initiation of biological agents was 15 (12–17) in patients in remission and was found to be statistically significantly higher (*p* = 0.004) compared to those not in remission [13 (10–15)]. Similar to the results in the first year, no significant differences were observed between the remission and non-remission groups in terms of pre-treatment attacks, OCS use, and number of hospitalizations (*p* > 0.05), while the median (IQR) serum total IgE value was again significantly higher in patients in remission compared to those not in remission (*p* = 0.015). The peripheral blood eosinophil count prior to treatment was significantly higher in patients who had been in remission for the past year [250.5 (125–640) cells/µL] compared to those who had not been in remission [199 (93–305) cells/µL] (*p* = 0.027). No significant differences were found in respiratory function test parameters based on remission status (*p* > 0.05). There was no significant difference between the three groups based on asthma inflammation type (*p* = 0.211). The median (IQR) length of biological treatment was 48 (29–92) months for those in remission and 45 (32–84) months for those not in remission; there was no significant difference (*p* = 0.859)

When comorbidities were examined in terms of their relationship with the clinical remission status assessed in the final year of follow-up (Table 3), psychiatric illness and OSAS were observed more frequently in patients who did not achieve remission. While remission was not achieved in any of the patients with OSAS (n = 15), psychiatric illness was detected in 41.1% of patients who did not achieve remission and in 12.9% of patients who achieved remission. Both variables maintained their significant relationship with remission status after Holm–Bonferroni multiple test correction (*p* < 0.001). Arterial hypertension was more frequently observed in patients who did not achieve remission (56.7%) compared to those who achieved remission (40%) and showed a nominal association with remission status in unadjusted analyses (*p* = 0.036). However, since arterial hypertension was not a priori determined and lost its significance after multiple testing correction, this finding was considered exploratory. No significant association was found between other comorbidities and remission status in the last year assessment.

The relationship between patients’ pre-treatment eosinophil count, baseline ACT score, gender, and presence of psychiatric illness and achieving remission in the current year was evaluated using binary (binomial) logistic regression analysis (Table 4). The analysis revealed that these variables were significantly associated with remission. Female gender increased the likelihood of achieving remission by approximately 2.8 times (OR = 2.835; *p* = 0.042). A high baseline ACT score significantly increased the likelihood of achieving remission (OR = 1.141; *p* = 0.015). This result indicates that better asthma control at baseline positively influenced treatment response. Each 100 cells/µL increase in eosinophil count was significantly associated with increased odds of the outcome (OR = 1.179; *p* = 0.004). This finding suggests that higher baseline eosinophil levels may influence the treatment outcome. On the other hand, the presence of psychiatric illness was identified as the strongest negative determinant. The likelihood of achieving remission in individuals with psychiatric illness was approximately 5.4 times lower than in those without (OR = 5.389; *p* < 0.001).

In the subgroup analysis according to the biological agent used, among patients receiving mepolizumab therapy (n = 23), the remission rate was 91.3% at the end of the first year and 56.5% in the most recent year of follow-up. In the mepolizumab group, the mean FEV_1_ value was significantly higher in patients who remained in remission during the last year [2002 ± 637 mL] compared with those who were not in remission [1600 ± 1120 mL] (*p* = 0.047). Similarly, in the first year of mepolizumab treatment, the FEV_1_/FVC ratio was significantly higher in patients who achieved remission [79.5 ± 7.9%] compared with those who did not [65 ±14.5%] (*p* = 0.011).

In the omalizumab group, the remission rate was 54.7% at the end of the first year and 41.6% in the most recent year of follow-up among patients receiving omalizumab therapy (n = 137). Among patients who achieved remission, baseline ACT scores were significantly higher both at the end of the first year [14.1 ± 3 vs. 12.1 ± 3.4, *p* < 0.001] and in the most recent year [13.9 ± 3.4 vs. 12.8 ± 3.2, *p* < 0.001]. Additionally, serum total IgE levels were significantly higher in patients who achieved remission in the last year (*p* < 0.005). When comorbid conditions were evaluated in the omalizumab group, it was observed that remission was not achieved during the first year in 70% of patients with psychiatric disorders and 73.7% of those with osteoporosis (*p* < 0.001 and *p* = 0.007, respectively). Moreover, 80% of patients with psychiatric comorbidities remained without remission in the most recent year (*p* < 0.001).

Since the number of patients receiving Mepolizumab is much smaller, no comparison based on biological drugs was made.

## 4. Discussion

With the introduction and increasing use of biological therapies in severe asthma, clinical remission has become an important treatment goal today. Initially, this cohort was a typical example of a population with severe asthma, characterized by type-2 asthma phenotype, a high exacerbation rate, and a high symptom burden, along with significant corticosteroid use. With the use of biological therapy, after 1 year and last year, acute exacerbations and emergency department visits decreased significantly, symptom control improved, and a notable proportion of our patients achieved clinical remission.

When we looked at demographic characteristics, clinical remission rates in our study did not differ according to gender and age variables. This finding is consistent with other studies in the literature [9]. The natural decline in lung function and the effect of factors such as gender, age, smoking, BMI, ethnicity, dust exposure, and menopausal status on this decline should be considered when evaluating lung function in the definition of clinical remission [12]. Considering the time-dependent biological variation and measurement variability of FEV_1_ in the severe asthma population, it prevents clinically insignificant small changes from unnecessarily narrowing the definition of remission. Therefore, like Thomas et al. [10], we considered a decrease of up to 5% in FEV_1_ as stabilisation in our study. According to this criterion, our clinical remission rate under biological therapy was 60.0% in the first year and 43.7% in the last follow-up year. Yeşilkaya et al. [13] defined remission as an improvement of more than 10% in projected FEV_1_ and reported a first-year remission rate of 72.97% in a study of 74 patients. McDowell et al. [11] defined remission as having FEV_1_ above the lower limit of normal or a drop of no more than 100 mL from baseline and observed a remission rate of 18% in a sample of 830 patients. Therefore, compared to existing data, our study shows high remission rates under biological treatments; however, the results should be interpreted with caution due to methodological differences.

Maintenance oral corticosteroid use has generally been associated with lower clinical remission rates, as demonstrated in a previous meta-analysis of biological treatments [3]. In our study, it was observed that patients receiving maintenance systemic corticosteroid therapy did not achieve remission at the end of the first year. On the other hand, remission rates were higher in patients with high ACT scores prior to treatment, consistent with the literature [13,14]. These findings suggest that patients who are clinically better and have less need for systemic steroids prior to biological therapy are more likely to achieve remission.

This study found that patients with high serum total IgE levels prior to biological treatment had higher remission rates. The fact that there were more patients receiving omalizumab treatment in our patient group may also partially explain this result. This finding supports the literature reporting that high IgE levels are associated with a better clinical response and remission likelihood, particularly with omalizumab treatment [15,16,17]. In our study, the remission rate was found to be higher in patients with elevated peripheral blood eosinophil levels prior to initiation of biological therapy. This finding supports the notion, as emphasized in the literature, that elevated blood eosinophil counts are a strong marker of biological response and clinical remission, particularly in anti-IL-5 therapies [6,9]. Various studies report higher clinical remission rates in patients receiving anti-IL-5 therapy compared to those receiving anti-IgE therapy [11,13].

Although a higher remission rate was observed among inflammatory phenotypes, the finding of a 100% remission rate in the non-allergic eosinophilic subgroup should be interpreted cautiously due to the small sample size (n = 8) and potential biological implausibility. This result is likely sample-dependent and should be considered exploratory rather than confirmatory. Larger studies are required to validate this observation.

The likelihood of clinical remission in asthma is closely related not only to the response to biological therapy but also to accompanying comorbid conditions. Various studies have shown that comorbidities complicate disease control, increase the risk of exacerbations, and may limit the response to biological therapies [18,19].

According to our results, asthma remission rates were found to be low in patients with psychiatric comorbidity, which is consistent with the literature. When evaluating possible mechanisms explaining this situation, the first is altered symptom perception. Asthma and depression symptomatology overlap significantly, which can lead to misinterpretation of symptoms and inappropriate escalation of treatment [20]. Therefore, even if lung function is objectively stabilized, achieving symptom-based clinical remission criteria may be difficult in individuals with psychiatric comorbidity due to “higher symptom reporting” [21]. Secondly, reduced adherence to treatment may be an important mechanism. In asthma, symptoms of anxiety and depression have been shown to be negatively associated with adherence to inhaler treatment; as adherence declines, symptom control worsens, and this is reflected in clinical outcomes [22]. The third and increasingly popular explanation is neuro-immune interactions. Psychological stress and mood disorders can modulate the immune response via the hypothalamic-pituitary-adrenal (HPA) axis, the autonomic nervous system, and cytokine networks. In this context, depression should be considered not as a comorbidity in asthma management, but as a factor that actively modulates asthma pathophysiology. Treatments that target inflammation alone are insufficient to achieve clinical remission in the presence of autonomic dysfunction [23]. When these findings are considered together, it is thought that psychiatric comorbidities may multidimensionally reduce the likelihood of clinical remission in asthma by affecting not only symptom perception but also treatment behaviors and underlying immune mechanisms.

Our results show that remission rates in OSAS patients were significantly lower, consistent with the literature. Studies by Teodorescu [24]. and Yigla [25] have shown that OSAS is associated with poorer symptom control, increased exacerbation frequency, and treatment resistance in patients with severe asthma. Therefore, in the evaluation of patients with severe asthma, not only airway inflammation and biomarkers but also the presence and severity of comorbidities should be considered. This approach will contribute to setting more realistic remission goals with biological therapies and planning more comprehensive patient management.

There are some limitations to our study. First, our patient sample size is relatively small, and the results should be interpreted with caution in terms of generalizability. The retrospective design of our study is another limitation. Retrospective data collection may have led to incomplete or non-standard recording of clinical information, potentially preventing the homogeneous assessment of certain parameters. Additionally, there was a significant imbalance in the number of patients receiving biological therapies; 137 patients were treated with omalizumab, while 23 patients were treated with mepolizumab. This disparity can be explained by the retrospective time frame of the study (2009–2023) and the varying levels of accessibility of these biological agents in our country. While omalizumab has been on the market since 2009, mepolizumab only entered clinical practice in 2019. The shorter duration of mepolizumab use contributed to the lower number of patients receiving this treatment. Furthermore, at the time the study received ethical approval, no other biological agents were approved for asthma treatment in our country, so additional biological treatments could not be included in the study. In addition, FEV_1_ measurements could not be obtained at regular or annual intervals during the follow-up period; spirometric assessments were only available in the first year of biological maintenance therapy and at the patients’ final follow-up visit. Therefore, longitudinal analyses and curve modeling reflecting the time-dependent long-term course of FEV_1_ could not be performed, and evaluations were limited to the available measurement time points.

On the other hand, the exclusion of patients who underwent biological therapy switching from the analysis is an advantage of our study. Indeed, as noted in the literature, it is difficult to assess long-term remission in patients who have undergone a switch, and therefore, many real-world studies exclude this patient group from their analysis [26,27]. The approach of not including switch cases in the study may have contributed to our relatively high remission rate. Furthermore, the earlier initiation and longer duration of omalizumab use compared to mepolizumab use in our country may have resulted in fewer patients receiving mepolizumab who had not previously received biological therapy.

## 5. Conclusions

In conclusion, a high rate of clinical remission can be achieved at the end of the first year in patients with severe asthma who start treatment with omalizumab or mepolizumab; however, this rate decreases as the follow-up period lengthens. A high ACT score and eosinophil level prior to treatment are positively associated with remission, while psychiatric disorders and OSAS negatively affect remission success. Mepolizumab treatment has been shown to provide higher remission in the early period, but this effect may decrease in the long term. These results show that biological treatments in severe asthma are promising in terms of clinical remission, but patient characteristics, comorbidities, and biological treatment selection must be carefully evaluated for sustainable remission. Long-term studies involving larger patient groups and different biological agents will fill the knowledge gap in this area.

## Figures and Tables

**Table 1:** Patient characteristics in the clinical remission assessment at the first year of maintenance biologic therapy.

	Total (n: 160)	Remission Present n: 96	Remission Absent n: 64	*p*
Age, years	53.76 ± 14.62	53.16 ± 14.9	54.67 ± 14.24	0.519
Gender				
Male	29 (18.1%)	16 (16.7%)	13 (20.3%)	0.706
Female	131 (81.9%)	80 (83.3%)	51 (79.7%)	
Body mass index, kg/m^2^	29 (25–34)	28.62 (24–33)	30 (27–34)	0.104
Smoking status				
Non-smoker	101 (63.1%)	62 (64.6%)	39 (60.9%)	0.403
Ex-smoker	47 (29.4%)	29 (30.2%)	18 (28.1%)	
Active smoker	12 (7.5%)	5 (5.2%)	7 (10.9%)	
Smoking, pock/years	0 (0–10)	0 (0–10)	0 (0–10)	0.526
Age at onset of asthma, years	40.10 ± 13.87	40.81 ± 14.12	38.98 ± 13.54	0.404
Duration of asthma, years	12 (8–17.5)	10 (8–16)	13 (8–22.5)	0.120
Atopy	152 (95%)	88 (91.7%)	64 (100.0%)	0.022 *
House dust mite	138 (86.3%)	78 (81.3%)	60 (93.8%)	0.044 *
Cockroach	32 (20%)	18 (18.8%)	14 (21.9%)	0.778
Mold	51 (31.9%)	30 (31.3%)	21 (32.8%)	0.972
Cat dander	19 (11.9%)	12 (12.5%)	7 (10.9%)	0.960
Latex	11 (6.9%)	9 (9.4%)	2 (3.1%)	0.202
Pollen	68 (42.5%)	45 (46.9%)	23 (35.9%)	0.227
Asthma inflammation type				
Allergic non-eosinophilic	27 (16.9%)	19 (19.8%)	8 (12.5%)	0.021 *
Nonallergic eosinophilic	8 (5%)	8 (8.3%)	0 (0%)	
Allergic eosinophilic	125 (78.1%)	69 (71.9%)	56 (87.5%)	
Comorbidities				
Nasal polyp	25 (15.6%)	19 (19.8%)	6 (9.4%)	0.120
Rhinosinusitis	156 (97.5%)	93 (96.9%)	63 (98.4%)	0.650
N-ERD	13 (8.1%)	9 (9.4%)	4 (6.3%)	0.679
Obstructive sleep apnea	15 (9.4%)	7 (7.3%)	8 (12.5%)	0.406
Gastroesophageal reflux	43 (26.9%)	23 (24.0%)	20 (31.3%)	0.402
Psychiatric disorder	46 (28.8%)	16 (16.7%)	30 (46.9%)	0.001 **
Osteoporosis	24 (15%)	9 (9.4%)	15 (23.4%)	0.027
Arterial hypertension	79 (49.4%)	45 (46.9%)	34 (53.1%)	0.619
Diabetes mellitus	37 (23.1%)	24 (25.0%)	13 (20.3%)	0.491
Bronchiectasis	20 (12.5%)	8 (8.3%)	12 (18.8%)	0.088
Immunodeficiency	4 (2.5%)	0 (0%)	4 (6.3%)	0.024
Obesity	131 (81.9%)	82 (85.4%)	49 (76.6%)	0.224
Before biologic therapy				
Exacerbation	144 (90%)	86 (89.6%)	58 (90.6%)	1.000
Exacerbation/year	2 (1–4)	2 (0–4)	2 (1–5)	0.146
Use of oral corticosteroids	108 (67.5%)	62 (64.6%)	46 (71.9%)	0.428
OCS courses/year	1 (0–2)	1 (0–2)	1 (0–3)	0.098
Continuous OCS use	3 (1.9%)	0 (0.0%)	3 (4.7%)	0.062
Hospitalization	35 (21.9%)	19 (19.8%)	16 (25.0%)	0.558
Hospitalization/year	0 (0–0)	0 (0–0)	0 (0–0.5)	0.392
Asthma Control Test	14 (12–16)	14 (12.5–16)	12 (10–15)	<0.001 **
Laboratory				
Total IgE, IU/mL	121.5 (51–286)	138 (65.5–320.5)	94.5 (45–186.5)	0.048 *
Eosinophil, cells/µL	212.5 (105–408.5)	234 (105.5–476.5)	188.5 (101–303.5)	0.129
Pulmonary function test				
FEV_1_, mL	2192.81± 863.12	2188.02 ± 819.2	2200 ± 931.71	0.932
FEV_1_% predicted	86.5 (65–100)	86 (64.5–98)	88.5 (66–104)	0.425
FVC, mL	2802.87 ± 1003.46	2831.35 ± 982.34	2760.16 ± 1040.71	0.665
FVC, % predicted	92.5 (75–102)	89 (73–102)	94 (75–103)	0.680
FEV_1_/FVC, % predicted	77.52 ± 10.70	76.81 ± 10.53	78.58 ± 10.94	0.312

*p* *: <0.05, *p* **: <0.01. Data are given as mean ± standard deviation or median (1st quartile—3rd quartile) for continuous variables and as frequency (percentage) for categorical variables. The *p*-values presented in the table have not been adjusted. Multiple testing adjustment (Holm–Bonferroni) has been applied for pre-specified comorbidities, and the adjusted findings are described in the Section 3. N-ERD: Nonsteroidal anti-inflammatory drug–exacerbated respiratory disease, OCS: Oral corticosteroid, Total IgE: Total Immunoglobulin E, FEV_1_: Forced expiratory volume in 1 second, FVC: Forced vital capacity.

**Table 2:** Multivariate logistic regression analysis of independent factors predicting clinical remission at the first year after biologic therapy.

	β Coefficient	Standard Error	*p*	Exp (B)	95% CI for EXP (B)	
					Lower	Upper
1 Asthma Control Test	0.221	0.059	<0.001	1.247	1.112	1.399
2 Eosinophil Count *	0.133	0.061	0.030	1.142	1.013	1.288
3 Psychiatric disorder	1.561	0.398	<0.001	4.763	2.184	10.386

CI: Confidence Interval, BMI: Body mass index, FEV_1_: Forced expiratory volume in 1 second, ACT: Asthma Control Test, Total IgE: Total Immunoglobulin E. Variables entered in Step 1: Age, Gender, BMI, FEV_1_ % predicted, ACT, Total IgE, eosinophil count, psychiatric disorder. * Eosinophil counts were grouped into 100 cells/µL intervals. Nagelkerke R^2^: 28.5%, Overall Percentage: 69.4.

**Table 3:** Patient characteristics in the clinical remission assessment at the latest year of maintenance biologic therapy.

	Total (n: 160)	Remission Present N: 70	Remission Absent N: 90	*p*
Age, years	53.76 ± 14.62	52.54 ± 14.16	54.71 ± 14.97	0.350
Gender				
Male	29 (18.1%)	10 (14.3%)	19 (21.1%)	0.365
Female	131 (81.9%)	60 (85.7%)	71 (78.9%)	
Body mass index, kg/m^2^	29 (25–34)	29.5 (25–33)	29 (25–35)	0.540
Smoking status				
Non-smoker	101 (63.1%)	45 (64.3%)	56 (62.2%)	0.383
Ex-smoker	47 (29.4%)	22 (31.4%)	25 (27.8%)	
Active smoker	12 (7.5%)	3 (4.3%)	9 (10.0%)	
Smoking, pock/years	0 (0–10)	0 (0–5)	0 (0–15)	0.434
Age at onset of asthma, years	40.10 ± 13.87	39.95 ± 12.84	40.24 ± 14.85	0.981
Duration of asthma, years	12 (8–17.5)	10 (8–17)	12 (8–18)	0.243
Atopy	152 (95%)	64 (91.4%)	88 (97.8%)	0.139
House dust mite	138 (86.3%)	61 (87.1%)	77 (85.6%)	0.954
Cockroach	32 (20%)	14 (20.0%)	18 (20.0%)	1.000
Mold	51 (31.9%)	18 (25.7%)	33 (36.7%)	0.192
Cat dander	19 (11.9%)	7 (10.0%)	12 (13.3%)	0.689
Latex	11 (6.9%)	4 (5.7%)	7 (7.8%)	0.757
Pollen	68 (42.5%)	31 (44.3%)	37 (41.1%)	0.687
Asthma inflammation type				
Allergic non-eosinophilic	27 (16.9%)	12 (17.1%)	15 (16.7%)	0.211
Nonallergic eosinophilic	8 (5%)	6 (8.6%)	2 (2.2%)	
Allergic eosinophilic	125 (78.1%)	52 (74.3%)	73 (81.1%)	
Comorbidities				
Nasal polyp	25 (15.6%)	15 (21.4%)	10 (11.1%)	0.118
Rhinosinusitis	156 (97.5%)	68 (97.1%)	88 (97.8%)	1.000
N-ERD	13 (8.1%)	6 (8.6%)	7 (7.8%)	1.000
Obstructive sleep apnea	15 (9.4%)	0 (0.0%)	15 (16.7%)	<0.001 **
Gastroesophageal reflux	43 (26.9%)	15 (21.4%)	28 (31.1%)	0.234
Psychiatric disorder	46 (28.8%)	9 (12.9%)	37 (41.1%)	<0.001 **
Osteoporosis	24 (15%)	8 (11.4%)	16 (17.8%)	0.372
Arterial hypertension	79 (49.4%)	28 (40.0%)	51 (56.7%)	0.036
Diabetes mellitus	37 (23.1%)	14 (20.0%)	23 (25.6%)	0.524
Bronchiectasis	20 (12.5%)	8 (11.4%)	12 (13.3%)	0.904
Immunodeficiency	4 (2.5%)	0 (0.0%)	4 (4.4%)	0.132
Obesity	131 (81.9%)	61 (87.1%)	70 (77.8%)	0.187
Before biologic therapy				
Exacerbation	144 (90%)	64 (91.4%)	80 (88.9%)	0.791
Exacerbation/year	2 (1–4)	2 (1–4)	2 (1–4)	0.678
Use of oral corticosteroids	108 (67.5%)	44 (62.9%)	64 (71.1%)	0.269
OCS courses/year	1 (0–2)	1 (0–2)	1 (0–2)	0.667
Continuous OCS use	3 (1.9%)	0 (0.0%)	3 (3.3%)	0.257
Hospitalization	35 (21.9%)	12 (17.1%)	23 (25.6%)	0.278
Hospitalization/year	0 (0–0)	0 (0–0)	0 (0–0)	0.260
Asthma Control Test	14 (12–16)	15 (12–17)	13 (10–15)	0.004 **
Laboratory				
Total IgE, IU/mL	121.5 (51–286)	152 (80–340)	107 (42–191)	0.015 *
Eosinophil, cells/µL	212.5 (105–408.5)	250.5 (125–640)	199 (93–305)	0.027 *
Pulmonary function test				
FEV_1_, mL	2192.81± 863.12	2115.71 ± 688.14	2252.78 ± 977.48	0.300
FEV_1_% predicted	86.5 (65–100)	84.5 (63–98)	88 (70–104)	0.284
FVC, mL	2802.87 ± 1003.46	2748.71 ± 834.25	2845.0 ± 1120.38	0.534
FVC, % predicted	92.5 (75–102)	88 (71–102)	94 (75–103)	0.607
FEV_1_/FVC, % predicted	77.52 ± 10.70	76.87 ± 10.35	78.02 ± 10.99	0.498
Biologic agent use duration, months	45 (29–81.5)	48 (29–92)	45 (32–84)	0.859

*p* *: <0.05, *p* **: <0.01. Data are given as mean ± standard deviation or median (1st quartile—3rd quartile) for continuous variables and as frequency (percentage) for categorical variables. The *p*-values presented in the table have not been adjusted. Multiple testing adjustment (Holm–Bonferroni) has been applied for pre-specified comorbidities, and the adjusted findings are described in the Section 3. N-ERD: Nonsteroidal anti-inflammatory drug–exacerbated respiratory disease, OCS: Oral corticosteroid, Total IgE: Total Immunoglobulin E, FEV_1_: Forced expiratory volume in 1 second, FVC: Forced vital capacity.

**Table 4:** Multivariate logistic regression analysis of independent factors predicting clinical remission at the latest year.

		β coefficient	Standard Error	*p*	Exp (B)	95% CI for EXP (B)	
						Lower	Upper
1	Gender	1.042	0.512	0.042	2.835	1.039	7.734
2	Asthma Control Test	0.132	0.054	0.015	1.141	1.026	1.268
3	Eosinophil count *	0.165	0.057	0.004	1.179	1.054	1.320
4	Psychiatric disorder	1.684	0.443	<0.001	5.389	2.262	12.841

CI: Confidence Interval, BMI: Body mass index, FEV_1_: Forced expiratory volume in 1 second, ACT: Asthma Control Test, Total IgE: Total Immunoglobulin E. Variables entered in Step 1: Age, Gender, BMI, FEV_1_%, ACT, Total IgE, Eosinophil count, Psychiatric disorder. * Eosinophil counts were grouped into 100 cells/µL intervals. Nagelkerke R^2^: 25.6%, Overall Percentage: 71.3%.

## Data Availability

The data are available from the corresponding author upon reasonable request due to ethical restrictions and data protection considerations.

## Abbreviations

ACQ	Asthma Control Questionnaire
ACT	Asthma Control Test
ATS	American Thoracic Society
BMI	Body Mass Index
CI	Confidence Intervals
ERS	European Respiratory Society
FeNO	Fractional Exhaled Nitric Oxide
FEV_1_	Forced Expiratory Volume
FVC	Forced Vital Capacity
GER	Gastroesophageal Reflux
GINA	Global Initiative for Asthma
HPA	Hypothalamic-pituitary-adrenal
IgE	Immunoglobulin E
NERD	Nonsteroidal anti-inflammatory drugs-Exacerbated Respiratory Disease
OCS	Oral Corticosteroid
OR	Odds Ratios
OSAS	Obstructive Sleep Apnea Syndrome
PEF	Peak Expiratory Flow
SD	Standard Deviation
